# The Effect of Maternal Diet and Lifestyle on the Risk of Childhood Obesity

**DOI:** 10.3390/metabo14120655

**Published:** 2024-11-25

**Authors:** Edyta Łuszczki, Justyna Wyszyńska, Agnieszka Dymek, Dorota Drożdż, Laura González-Ramos, Isa Hartgring, Nuria García-Carbonell, Artur Mazur, Serap Erdine, Justė Parnarauskienė, Julio Alvarez-Pitti

**Affiliations:** 1 Institute of Health Sciences, Medical College of Rzeszów University, 35-959 Rzeszów, Poland; jwyszynska@ur.edu.pl (J.W.); adymek@ur.edu.pl (A.D.); 2 Department of Pediatric Nephrology and Hypertension, Pediatric Institute, Jagiellonian University Medical College, 31-007 Krakow, Poland; dorota.drozdz@uj.edu.pl; 3 Innovation in Paediatrics and Technologies-iPEDITEC- Research Group, Fundación de Investigación, Consorcio Hospital General, University of Valencia, 46010 Valencia, Spain; lauragonzalezramos99@gmail.com (L.G.-R.); isahartgring@gmail.com (I.H.); nuria.garcia-carbonell@uv.es (N.G.-C.); japnago@gmail.com (J.A.-P.); 4 Pediatric Department, Consorcio Hospital General, University of Valencia, 46014 Valencia, Spain; 5 Institute of Medical Sciences, Medical College of Rzeszów University, 35-959 Rzeszów, Poland; drmazur@poczta.onet.pl; 6 Cerrahpasa Faculty of Medicine, Department of Cardiology, Istanbul University-Cerrahpasa, 34320 Istanbul, Turkey; serap.erdine@gmail.com; 7 Pediatric Department, Vilnius University Hospital Santaros Klinikos, 08661 Vilnius, Lithuania; juste.parnarauskiene@santa.lt; 8 CIBER Fisiopatología Obesidad y Nutrición (CIBEROBN), Instituto de Salud Carlos III, 28029 Madrid, Spain

**Keywords:** childhood obesity, fetal programming, epigenetics, maternal lifestyle, maternal diet, gut microbiota, gestational diabetes, obesogenes

## Abstract

**Background/Objectives**: Childhood obesity is a global health problem that affects at least 41 million children under the age of five. Increased BMI in children is associated with serious long-term health consequences, such as type 2 diabetes, cardiovascular disease, and psychological problems, including depression and low self-esteem. Although the etiology of obesity is complex, research suggests that the diet and lifestyle of pregnant women play a key role in shaping metabolic and epigenetic changes that can increase the risk of obesity in their children. Excessive gestational weight gain, unhealthy dietary patterns (including the Western diet), and pregnancy complications (such as gestational diabetes) are some of the modifiable factors that contribute to childhood obesity. The purpose of this narrative review is to summarize the most important and recent information on the impact of the diet and lifestyle of pregnant women on the risk of childhood obesity. **Methods**: This article is a narrative review that aims to summarize the available literature on the impact of pregnant women’s diet and lifestyle on the risk of obesity in their offspring, with a focus on metabolic and epigenetic mechanisms. **Results**/**Conclusions**: Current evidence suggests that a pregnant woman’s lifestyle and diet can significantly contribute to lowering the risk of obesity in their offspring. However, further high-quality research is needed to understand better the metabolic and epigenetic relationships concerning maternal factors that predispose offspring to obesity.

## 1. Introduction

Obesity rates are increasing worldwide every year, making it a major public health challenge. Obesity is not only a non-communicable disease but also a predisposing factor for many other conditions, including cardiovascular diseases, type 2 diabetes, mental health problems, and certain cancers [1]. It has been reported that in 2022, over 1 billion people suffered from obesity, and since 1990, the prevalence of obesity has more than doubled among adults and more than quadrupled among children and adolescents [2].

Over the past 50 years, childhood obesity has increased at a worrying rate, especially in developed countries. However, this phenomenon is also on the rise in developing countries. There is no doubt that obesity in the pediatric population is currently one of the most serious public health problems [3]. It is impossible to identify a single cause of childhood obesity, as the etiology of this condition depends on the reciprocal and complex interaction of several factors: behavioral; genetic; and environmental [3,4]. Today’s generations are growing up in an environment that is conducive to the development of obesity. There is a tendency to reduce physical activity in favor of a sedentary lifestyle and a marked increase in the consumption of high-calorie processed foods, leading to a positive energy balance and accumulation of body fat [5,6]. Despite the use of various weight loss strategies, long-lasting results are often not achieved, suggesting that weight control may not depend solely on willpower and consistent lifestyle changes. A growing body of scientific evidence indicates that the risk of childhood obesity may be influenced by an additional, equally significant factor—fetal metabolic programming [7,8,9]. The biological mechanisms that regulate energy homeostasis are established early in life, and their development is strongly influenced by conditions in the intrauterine environment [10]. In this context, maternal diet and lifestyle play a crucial role, as they can induce metabolic and epigenetic changes in the fetus, thereby affecting gene expression, organ development, and disease predisposition in the postnatal period [11].

In this review, we aim to explain how maternal diet and lifestyle may influence the risk of developing childhood obesity. We examine this relationship in the context of metabolic and epigenetic mechanisms, starting with an introduction to the concepts of fetal metabolic and epigenetic programming. Current research indicates that maternal nutrition is the most important factor affecting fetal programming [12,13,14]. Therefore, in the next section of this paper, we analyze how maternal nutritional status disorders (such as malnutrition and excessive body weight), dietary patterns (such as the Mediterranean and Western diets), and gut microbiota—which plays a significant role in the context of daily diet—may impact the offspring’s obesity risk. Food available in stores is often exposed to chemicals such as bisphenol A and phthalates, which are classified as obesogens; thus, we also explore this aspect [15]. We further consider gestational diabetes, a common pregnancy complication, as numerous studies suggest that this condition significantly impacts fetal metabolic programming [5].

## 2. Materials and Methods

This article is a narrative review that aims to summarize the available literature on the impact of pregnant women’s diet and lifestyle on the risk of obesity in their offspring, with a focus on metabolic and epigenetic mechanisms.

The literature search was conducted in databases such as Google Scholar, PubMed, Web of Science, Scopus, and Cochrane using a combination of the following keywords: “pregnancy”; “epigenetics”; “DNA methylation”; “histones modifications”; “non-coding RNAs”; “fetal programming”; “metabolic programming”; “epigenetic programming”; “maternal diet”; “maternal lifestyle”; “gut microbiota”; “gestational diabetes”; “obesogenes”; “environmental toxins”; “childhood obesity”; “diet”; “nutrition”; “prenatal nutritional physiological phenomena”; “epigenomics”; “metabolic factors”; “epigenetic modifications”; “metabolic pathways”.

This review primarily included publications from 2014 to 2024 to ensure the relevance of the research presented. In some cases, older studies were cited when they were essential for a complete and accurate description of the issues discussed. Additionally, to obtain a more comprehensive literature review and identify significant papers that may have been missed during database searches, the bibliographies of selected articles were also searched, which allowed for the discovery of additional important sources.

The inclusion criteria were as follows:

- Articles published in the last ten years (between 2014 and 2024);
- Article types, such as meta-analyses, reviews, systematic reviews, clinical studies, observational studies, animal studies, human studies, recommendations, guidance documents, and books;
- Articles with full-text access;
- Articles published in English;
- Articles examining the influence of prenatal factors on fetal metabolic and/or epigenetic programming;
- Articles examining fetal metabolic and/or epigenetic programming in relation to maternal diet and lifestyle (maternal nutritional status, dietary patterns such as the Mediterranean and Western diets, gut microbiota, obesogens, and gestational diabetes).

**Figure 1:**
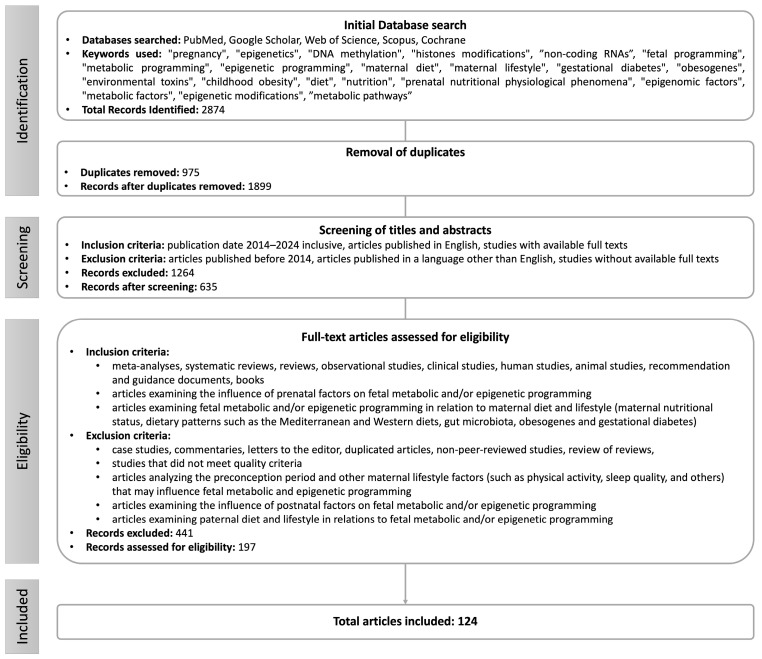
Methodology selection flowchart.

Exclusion criteria included the following:

- Articles published before 2014;
- Article types, such as case studies, commentaries, letters to the editor, non-peer-reviewed articles, and reviews of the reviews;
- Articles without access to full text;
- Articles published in a language other than English;
- Articles examining the preconception period and other maternal lifestyle factors (such as physical activity, sleep quality, and others) that may influence fetal metabolic and epigenetic programming;
- Articles examining the influence of postnatal factors on fetal metabolic and/or epigenetic programming;
- Articles examining paternal diet and lifestyle in relation to fetal metabolic and/or epigenetic programming;

The search process for this narrative review is illustrated in Figure 1.

## 3. Fetal Metabolic and Epigenetic Programming

### 3.1. Fetal Metabolic Programming

Metabolic programming (also known as fetal programming) is the concept that the intrauterine environment, modulated by external factors, including diet, can induce phenotypic changes in the child’s body during so-called critical periods of development [16]. This may result in metabolic and functional changes in organs, which, in turn, may predispose the offspring to chronic diseases. Many factors shape the intrauterine environment [17]. The term ‘nutritional programming’ is used in the context of nutritional factors. According to this theory, inappropriate maternal nutrition (both over- and under-nutrition) during the first 1000 days of fetal life can cause irreversible changes in the child’s organs and metabolism [18]. The concept of nutritional programming was pioneered by the British physician and epidemiologist David Barker, whose observations in the 1990s led him to speculate that the experience of fetal starvation might be an early source of cardiovascular and metabolic diseases, possibly due to fetal programming [19]. According to the ‘Barker hypothesis’, malnutrition during this critical development period can cause irreversible changes in the structure and function of organs such as the pancreas, liver, and kidneys, in favor of normal brain function [20]. In this way, the fetus is able to survive by adapting to a nutritionally deficient environment. However, this can have negative consequences, as after birth, the infant will be faced with an environment where food (especially high-calorie food) is readily available, unlike the prenatal period. In such a situation, the infant’s body may find it difficult to adapt to the excess calories, making it more susceptible to the development of obesity and other chronic metabolic diseases [19,21].

Although maternal nutrition appears to be the most important determinant of fetal intrauterine programming [22], recent studies in humans and animals have provided substantial evidence for other factors that may program a child’s metabolism and predispose them to obesity. These factors include maternal overall physiology (e.g., the presence of obesity [23,24,25]), complications during pregnancy (e.g., gestational diabetes [26,27,28]), maternal gut microbiota [29,30], and exposure to chemicals classified as obesogens, such as bisphenol A (BPA) and phthalates [31,32,33,34]. Metabolic programming is a complex process, and the mechanisms triggered by an adverse intrauterine environment are poorly understood. However, much evidence suggests that epigenetic factors play an important role in this process [17,35].

### 3.2. Fetal Epigenetic Programming

Epigenetics is a branch of science that studies changes in gene expression (both stable and heritable) that result from changes in chromatin structure rather than changes in the sequence of the genetic code [36]. Epigenetic mechanisms affect the regulation of gene expression and result in their activation or silencing, depending on the tissue type [37]. This is important in cell differentiation and the acquisition of a specific function [38]. The human epigenome is modified early in life due to the pre- and perinatal environments. This has a profound effect on gene expression throughout life [39]. However, this does not mean epigenetic changes do not occur in adulthood. Studies of monozygotic twins suggest that exposure to environmental factors later in life can alter human epigenetic patterns [40,41].

As mentioned above, epigenetics plays a key role in fetal metabolic programming. The intrauterine environment can induce changes in the epigenetic patterns of the developing organism by influencing gene expression. These changes are replicated during numerous cell divisions and remain stable into adulthood. As a result, they define the child’s phenotype and can predispose the child to certain diseases, including obesity. This phenomenon is known as fetal epigenetic programming [10,17].

The main mechanisms underlying epigenetic modifications include DNA methylation, post-translational modifications of histones, and non-coding RNAs (Table 1) [42].

#### 3.2.1. DNA Methylation

DNA methylation involves the addition of a methyl group to the fifth carbon atom in the cytosine nucleotide ring at the CpG (cytosine–phosphate–guanine) dinucleotide-rich DNA site. This reaction is catalyzed by DNA methyltransferases and results in the formation of 5-methylcytosine [43]. The donor of the methyl group is S-adenosylmethionine (SAM), the levels of which are regulated by the diet, in particular by the intake of components such as vitamins B2, B6, B12, folic acid, and choline [44].

Depending on where the CpGs are located (enhancer region, promoter region, intragenic region), the effect of DNA methylation on the control of transcription varies [45]. The effect of DNA methylation is thought to silence gene expression by reducing the availability of DNA for transcription factors. However, current research suggests that hypermethylation of certain regions of the DNA strand can lead to gene transcription. This makes it difficult to formulate clear rules about the relationship between DNA methylation and gene transcription [46]. When methylation affects a promoter or enhancer region, gene repression is observed. In contrast, gene expression occurs with intragenic hypermethylation and promoter hypomethylation [42,43].

As mentioned above, cytosine methylation occurs mainly at CpG dinucleotides. However, these dinucleotides have the ability to cluster together to form CG-rich regions called CpG islands (CGIs), which are located near gene promoters. Unlike individual CpG dinucleotides, these islands are not usually methylated. This feature is evolutionarily conserved and promotes active gene expression [47].

DNA methylation is a process of particular importance in the context of human development. It occurs throughout life, but the most important changes occur during early embryonic development [12]. Initially, the DNA of the parental gametes is highly methylated. After fertilization, both before embryo implantation and after implantation in the uterus, methylation of embryonic DNA is reprogrammed. Most of the methylation patterns inherited from the mother and father are removed and then re-established and duplicated in the somatic cells. These processes are crucial for establishing a methylation pattern that is unique to the embryo [48]. However, there is a group of genes that do not undergo this reprogramming during embryonic development. These are known as ‘imprinted’ genes. Genetic imprinting is the process by which one of the two alleles of a gene (inherited from the mother or father) is silenced, depending on its origin. This is the result of differences in DNA methylation that occur during the formation of reproductive cells (oocytes or sperm) [49]. Most imprinted genes play a key role in fetal development, growth, and metabolism, and imprinting disorders can lead to various diseases, such as Prader–Willi syndrome or Angelman syndrome [50,51,52].

#### 3.2.2. Post-Translational Modifications of Histones

Histones are proteins involved in regulating the structure and condensation of chromatin. When chromatin adopts a relaxed configuration (euchromatin), transcription is possible. When chromatin adopts a compact, condensed configuration (heterochromatin), transcription is not possible, and consequently, gene expression does not occur [53,54]. Post-translational modification of histones is another epigenetic mechanism that affects the activity of genomic regions. This occurs in two ways: by altering chromatin structure and by affecting the attachment of chromatin-associated proteins [55,56,57]. Histone proteins can be modified in many ways, including methylation, acylation (e.g., acetylation), phosphorylation, ubiquitination, sumoylation, or ADP-ribosylation [55,58,59]. Phosphorylation and acetylation of histones cause chromatin relaxation, allowing for active transcription. Methylation, in turn, can activate or inhibit transcription, depending on the methylated residue. For example, the addition of a methyl group to histone H3 arginine 17 (H3R17), lysine 4 (H3K4), or lysine 36 (H3K36) activates transcription. In contrast, transcription is repressed when histone H3 is methylated at lysine 9 (H3K9) or histone H4 is methylated at lysine 20 (H4K20) [60,61].

Previous published work suggests that there is an interaction between histone modifications and DNA methylation [62,63]. The MeCP2 protein binds to methylated CpG sequences and recruits proteins such as histone deacetylases (HDACs) and histone methyltransferases, leading to modifications within histones. In turn, histone-modifying enzymes, such as HDAC1 and HDAC2, and components of the polycomb repressive complex 2 can recruit DNMT1, which is responsible for adding methyl groups to DNA [12].

#### 3.2.3. Non-Coding RNAs

Although 90% of the human genome is transcribed, less than 2% of RNAs encode proteins. The remaining RNAs are non-coding RNAs (ncRNAs), which are not translated into proteins but are involved in key mechanisms that regulate the reading of genetic information [64]. Depending on their length, there are two main types of ncRNAs: short (<200 nucleotides) ncRNAs (miRNA, piRNA, siRNA) and long (>200 nucleotides) ncRNAs, which include eRNA, ilncRNA, and lincRNA [65,66,67]. Gene expression regulation by ncRNA occurs at transcriptional, post-transcriptional, and translational levels through various mechanisms, including chromatin remodeling, RNA interference, modulation of DNA methylation, and histone modifications [12,68,69].

Early embryonic development is characterized by global changes in the epigenome in response to conditions in the intrauterine environment. During this period, the embryo exhibits a high degree of plasticity and sensitivity to external factors, such as feeding patterns and maternal lifestyle, which can influence epigenetic and metabolic modifications and subsequent fetal development [70,71]. Epigenetic modifications alter the way genetic information is read without affecting the DNA sequence. They, therefore, affect many biological processes in the child’s body, including susceptibility to metabolic disorders [72]. In the context of obesity, it is particularly relevant that changes in the fetal epigenome can affect genes involved in energy metabolism, glucose regulation, insulin signaling, or processes related to adipogenesis [73].

## 4. Maternal Nutrition and Risk of Obesity in Offspring

The diet during pregnancy must provide the calories necessary to maintain the proper development of the fetus in the womb, the structures necessary for its maintenance, and to ensure the proper progress of the pregnancy. The mother’s diet during pregnancy cannot pose a risk, either by excess or deficiency, to the proper growth of the baby. Unfortunately, an unhealthy mother’s diet during pregnancy can even favor the development of chronic diseases at an early age, such as childhood obesity. This is characterized by a chronic inflammatory state that could be promoted by the mother’s diet, favoring weight gain in the newborn.

Maternal feeding affects fetal programming through epigenetic modifications. In addition, the diet followed during pregnancy is an epigenetic determinant that can modify the risk of chronic diseases in offspring. These could include cardiovascular diseases and obesity during childhood without modifying the genetic content. Dietary excess or deficiency may be related to the conditions that form the metabolic syndrome, leading to inflammation by modification of some genes, such as GNASAS, ABCA1, and IGF2. The same would happen in the case of a protein deficiency during pregnancy, which could affect the expression of genes related to fetal growth and glucose assimilation in the offspring. For this reason, the mother’s diet is the main focus of recent research [74].

The changes produced by pregnancy itself lead to an endocrine–metabolic adaptation favoring transient insulin resistance and a reduction in blood glucose, which is counteracted by an increase in glucose production by the liver. These physiological changes are essential to maintain fetal development during gestation [75]. In addition, these changes can affect the maternal metabolism of the different macronutrients present in the diet and their assimilation by the body.

The subject of maternal fat intake during pregnancy is a topic of ongoing debate. Evidence indicates that the consumption of fats during this period does not significantly influence the adiposity or body mass index (BMI) of offspring during their initial years of life. This conclusion remains consistent even in cases where there is supplementation with omega-3 fatty acids, specifically, eicosapentaenoic acid (EPA) and docosahexaenoic acid (DHA) [76,77]. However, in the study by Vidakovic A J et al., they found that plasma PUFA levels during pregnancy could influence the body composition of the offspring analyzed at 6 years of age. Specifically, higher omega-3 concentrations are associated with lower body fat percentage and android/gynoid ratio. Conversely, higher omega-6 values were associated with higher body fat percentage, android/gynoid ratio, and abdominal preperitoneal fat mass. Supporting this finding, if the mother had a higher n-6/n-3 ratio, this was associated with higher total and abdominal fat mass in the offspring [78]. This raises the idea that the anti-inflammatory effect of omega-3 fatty acids may have some protective effect on adiposity in children [76]. The study conducted by Hu Z et al. of 1257 mother–infant dyads during the second trimester of pregnancy provides insight into two common eating patterns in the US that are high in fat: fast food and processed food. Fast food consumption favors higher consumption of fried foods and sugary beverages, which are associated with higher fat intake and lower intake of available micronutrients. This leads to fetal development, promoting a more rapid increase in BMI in early childhood and the development of childhood obesity [79].

Higher protein intake during pregnancy is not consistently associated with changes in BMI or fat mass in offspring [80] but may have relevance for increased fat-free mass in 6-year-old children [81]. In the latter study, the results are obtained without showing differences by vegetable or animal origin of the protein. However, protein requirements during pregnancy are increasing, being higher in the second and third trimesters, according to the consensus of the different European guidelines [82]. Therefore, protein consumption by the pregnant woman should increase to guarantee adequate fetal development. Still, there is no evidence as to whether it affects the fat-free mass or the fat mass of the offspring, and studies in this direction are needed.

In line with the recommendation of the World Health Organization (WHO) to reduce consumption of simple sugars, maternal intake of sugars greater than 25 g (5% of total diet) [83] in healthy mothers appears to be associated with a 0.07 (95% CI: 0.01, 0.13) greater increase in BMI and a 0.02/mo (95% CI: 0.01, 0.03/mo) higher rate of weight gain in offspring aged 2–4 years [80]. Maintaining lower sugar and carbohydrate intake during late pregnancy, with increased protein and fat intake, appears to reduce the likelihood of having a higher fat percentage in preschoolers [76]. Considering glycaemic load, a low glycaemic index diet (GI ≤ 55) versus a moderate glycaemic index diet (GI around 60), maintaining the proportion of macronutrients in women at risk of diabetes, leads to offspring with lower weight, height, and arterial wall thickness, with no differences in adiposity [84]. In the same direction, another study from the Netherlands shows that overweight or obese women who consume high glycaemic index foods during the first trimester of pregnancy are associated with increased total, abdominal, and visceral fat in offspring [85]. On the other hand, although the average maternal carbohydrate intake remains unclear, there may be a relationship between higher maternal carbohydrate intake and lower fat percentage and fat mass in children without taking into account the glycaemic index and type of carbohydrate [86]. Given these results, the accuracy of carbohydrate and glycaemic index measurement, timing of pregnancy, and types of carbohydrates could be considered for future research in the prevention of childhood obesity.

Sugars are not the only nutrients that can have a bearing on children’s weight and growth; the diet as a whole can also play a role. For this, we can look at two known dietary patterns: the Mediterranean diet (MD) and the Western diet (WD). The main characteristics are shown in Table 2.

It has been found that there is a lack of scientific evidence analyzing the effect of the WD during pregnancy on offspring. This may be due to the fact that no studies have been conducted on this diet. However, as mentioned above, there is evidence linking the consumption of certain foods or nutrients during pregnancy with an impact on the cardiometabolic risk of the child in infancy. Examples of these foods could be pro-inflammatory foods, such as those rich in simple sugars and red meat. It is precisely these foods, among others, that characterize a WD or a diet considered to be pro-inflammatory. In the study by Sen S et al., the dietary inflammatory index (DII) was measured, which indicated how pro-inflammatory the diet was. Considering this value both during pregnancy and early childhood, it has been found that only in boys, an elevated DII was associated with higher BMI (adjusted *β* = 0.16 units per unit DII, 95%CI 0.02, 0.29), waist circumference (0.93 cm; −0.07, 1.92), and skinfolds (1.12 mm; 0.01, 2.23) [88]. Following this dietary pattern, a large cohort of 1459 mother–child dyads in Boston, Massachusetts, USA, showed that maintaining a diet with a higher inflammatory index and further away from a MD could increase weight in offspring during infancy and in children up to 10 years of age, independent of maternal weight [89]. A similar study analyzing adherence to these dietary patterns, after adjusting for maternal–child confounding factors (such as maternal age, education, or smoking), showed a trend, although not significant, of increased risk of childhood obesity in children at 1 and 3 years born to mothers following a Western diet [90].

In contrast, the MD stands out as a heart-healthy diet and appears to be the most suitable diet during pregnancy for offspring, characterized by increased fibre, antioxidants, micronutrients, and lower intakes of fat and sugars. In two cohorts, in the USA and Greece, greater adherence to the Mediterranean diet by pregnant women reduces blood pressure, leptin, and adiposity levels in offspring [91]. Moreover, in the INMA cohort in Spain, the growth pattern of offspring from birth to 4 years of age was analyzed. The mother’s diet had no impact on the cardiometabolic risk of their children by assessing DBP, SBP, HDL, and TG values. However, greater adherence to the MD was found to be associated with a lower risk of a larger infant at birth as well as a lower risk of an accelerated growth pattern [92]. The Accelerated Postnatal Growth Hypothesis is one of the assumptions regarding the likely causes of the early origins of increased body fat content and its derivations, such as childhood obesity or other pathologies [93,94]. Within the same cohort, no association was found between adherence to the MD during pregnancy and being overweight or obese at 4 years of age. However, greater adherence to the MD was inversely related to waist circumference, which is a marker of obesity and cardiovascular risk [95].

The benefits of the maternal MD during pregnancy are due to the modification of the expression of different genes through DNA methylation, histone modifications, non-coding RNAs, and microRNAs developed in different genes involved in comorbidities and the risk of obesity. Examples of these genes could be the CLOCK gene related to polyunsaturated fat consumption or the X retinoid-α receptor promoter region with low carbohydrate consumption. There are very few studies reflecting the effect of the mother’s Mediterranean dietary pattern on epigenetic changes in the offspring, but multiple references describe the impact of different nutrients on each of the mechanisms underlying epigenetic modifications [48,96,97]. The explanation of the interactions of each of these nutrients on the different mechanisms is beyond the scope of this review.

However, a new study has recently been published that attempted to pool a group of mother–child dyads, in which the degree of adherence to the Mediterranean diet and epigenetic changes in the cord blood of the offspring have been assessed. Increased adherence to the Mediterranean diet (MDA) has been shown to lead to beneficial changes in gene methylation in CpG regions, such as NCK2 related to adiposity and glucose tolerance. Moreover, placental methylation in the LIM domain only 3 (LMO3) is also associated with these changes in adiposity [98]. In addition, in a recent meta-analysis of epigenome-wide association studies (EWAS) in 2802 mother–child pairs from five cohorts in the Pregnancy And Childhood Epigenetics Consortium [71], methylation of cord blood samples at a specific CpG called cg23757341 related to the transcription of the WNT5B gene was highlighted. Overexpression of this gene could affect both adipogenesis and the development of type 2 diabetes.

## 5. Gestational Diabetes and Childhood Obesity

When analyzing the causes of the growing incidence of obesity in children and adults, attention was paid to the role of the course of pregnancy, the mother’s health, and pregnancy complications, including gestational diabetes mellitus (GDM) [99,100].

Hyperglycemia in pregnancy is associated with increased fetal growth, and it is hypothesized that fetal growth might be a mediator between GDM and overweight in the offspring. In a recent longitudinal cohort study of 6794 mother–child pairs, 295 presented with GDM, a higher fetal growth rate, and a higher risk of being overweight at ages 5 to 9 years in offspring of women with GDM [101].

Studies on the impact of gestational diabetes on the development of obesity in children have shown conflicting results regarding the relationship between GDM and obesity. However, research published in recent years indicates a relationship between GDM and an increased risk of developing obesity in children over 6 years of age. In one large clinical study (15,710 mother-offspring pairs), GDM was not associated with the development of early childhood obesity, but maternal obesity or overweight before pregnancy was independently associated with an increased risk of overweight in children at 2 years of age [102]. Li and co-authors examined the association between maternal GDM and the risk of obesity in offspring aged 6 to 8 years. They found a higher risk of these children developing overweight/obesity, central obesity, and high body fat. It should be emphasized that this significant and positive association became stronger with age [103]. Another interesting study investigated the associations of the timing of GDM diagnosis and gestational weight gains (GWG) with the growth trajectory of children from perinatal to early childhood. In a group of 7609 participants, a diagnosis of GDM before the 26th week of pregnancy was associated with an increased risk of greater abdominal circumference and fetal birth weight, as well as a higher maximum BMI at 48 months [104].

It has been suggested that considering metabolic factors in pregnant women, such as dyslipidemia and insulin resistance, compared to classifying women based on pre-pregnancy obesity or gestational diabetes, may improve obesity risk stratification in early childhood [105].

Offspring exposed to gestational diabetes mellitus in utero also have an increased risk of developing metabolic syndrome compared to those not exposed to GDM in utero (RR 2.07, 95% CI 1.26–3.42) [106]. The results of longitudinal cohort studies indicate the role of pre-pregnancy obesity in the development of GDM and its impact on body composition in offspring. Some authors point out that the effects of maternal pre-pregnancy overweight appear more pronounced [107]. The group of Hockett C studied BMI trajectories among youth exposed or not exposed to maternal GDM and found that children who are exposed to GDM may experience higher BMI trajectories during middle childhood and adolescence but not during early childhood [108].

Evidence from clinical and epidemiological studies suggests that maternal exposure to obesity or gestational diabetes in utero may increase the risk of overweight and obesity in children and adolescents.

## 6. The Maternal Gut Microbiome During Pregnancy and Its Role in Child Health

Contrary to the previously held notion of a “sterile placenta”, recent research has revealed the presence of microbiota in the placenta, amniotic fluid, umbilical cord blood, and meconium in healthy pregnancies [109]. The in utero colonization theory posits that the developing fetus is colonized by the low biomass placental microbiome during gestation [110]. This suggests that the infant’s microbiota may be acquired from the mother’s microbiome present in the in utero environment prior to birth [111]. Yang et al. have demonstrated that the offspring’s microbiome is influenced by genetic and intrauterine factors [112]. According to the study by Li et al., evidence supports the notion of a dysbiosis microbiome being vertically transmitted across the placenta to colonize the fetus before birth. This proposed pathway may contribute to the cycle of transgenerational obesity through microbial dysbiosis [113]. However, the precise mechanism of transmission remains ambiguous and necessitates further research.

Maternal BMI may potentially trigger placental inflammatory pathways through immune cells and pro-inflammatory cytokines. This indicates that higher maternal BMI associated with obesity can result in elevated pro-inflammatory cytokines in maternal plasma and activation of placental inflammatory pathways, potentially impacting placental function and leading to reduced oxygen levels and restricted blood flow, which can lead to adverse outcomes. Studies have also shown that the placentas of women with obesity exhibited an excessive inflammatory response involving macrophage infiltration via monocyte chemoattractant protein (MCP)-1 and tumor necrosis factor-alpha. It has been suggested that this impacted placental nutrient transfer negatively, as evidenced by decreased expression of sodium-dependent neutral amino acid transporters in the placentas of obese women. Furthermore, in vitro studies have indicated that IL-1β is inhibited by insulin-stimulated System A amino acid uptake in human placental trophoblast cells. Additionally, women with obesity have been found to have placental dysregulation of redox balance, reduced fatty acid transport, and increased placental lipoprotein lipase activity, potentially leading to placental triglyceride accumulation [114].

During pregnancy, hormonal changes can significantly impact the gut microbiota of both the mother and the infant. Research by Nuriel-Ohayon et al. has revealed that elevated progesterone levels in late pregnancy directly correlated with increased levels of Bifidobacterium in women and mice [115]. Bifidobacterium plays a vital role in breaking down human milk oligosaccharides (HMOs) found in maternal milk, which is crucial for infant nutrition in the neonatal period [116]. This suggests that an increase in Bifidobacterium in mothers may enhance the transmission of this beneficial genus to infants, aiding in the breakdown of HMOs and contributing to the development of the infant’s gut microbiota and immune system [117]. Guilley et al. conducted an examination of maternal HMOs in both mothers with elevated BMI and mothers with normal BMI, revealing a discernible alteration in their composition. Furthermore, progeny of obese mothers exhibited a diminished prevalence of short-chain fatty acid (SCFA)-producing bacteria and reduced levels of fecal butyric acid, thereby indicating an anticipated increase in adiposity at 12 months [118]. This underscores the potential for maternal obesity to imprint a predisposition for enduring obesity and concomitant metabolic and psychological ailments in offspring [10,119,120].

In murine models, it has been demonstrated that alterations in the maternal gut microbiota during pregnancy were correlated with maternal dietary patterns both prior to and during gestation [121]. Not only did the prenatal diet exert significant repercussions on the maternal microbiome, but maternal macronutrient intake has also shown a pronounced association with the neonatal gut microbiome in a human cohort comprising 86 mother–neonate pairs [122]. Additionally, it has been evidenced that both pre-pregnancy body weight and weight gain during pregnancy are linked to maternal modifications in the gut microbiota. A study encompassing 169 women revealed that pre-pregnancy overweight or obese status was linked to diminished alpha diversity and variations in microbial composition, while excessive gestational weight gain showed an association with compositional disparities in the gut microbiome [123].

The impact of obesity development extends beyond infancy and manifests lifelong implications. Children with obesity exhibit heightened metabolic activity in their gut microbiota compared to their lean counterparts, contributing to the etiology of persistent obesity. This increased metabolic activity is quantified by the presence of active enteric bacteria and the volume of SCFAs generated [124]. Notably, alterations in microbial compositions are evident in children with obesity, even at the species level [125]. For instance, the likelihood of Neisseria carriage is three times higher in children with obesity compared to non-obese individuals, with the risk escalating with greater BMI severity [126]. These findings underscore the enduring influence of the maternal microbiome on offspring, highlighting the detrimental impact of an obesogenic and dysbiosis maternal microbiome on offspring health.

The delivery method also impacts the colonization of the human infant gut microbiome. Vaginal delivery impacts the infant’s intestinal bacteria by introducing vaginal species, while this process is disrupted during cesarean section (C-section) [127]. Multiple human studies have demonstrated delayed colonization of the gut by species such as Bacteroides, Lactobacillus, Bifidobacterium, and Bacteroidetes in infants born by C-section [128]. Reyman et al. found that infants born vaginally had notably higher levels of Bifidobacterium at 1 week, which was associated with positive health outcomes [129]. In a study involving 82 newborns, infants born through C-section showed significantly higher levels of Staphylococcus and Streptococcus genera compared to vaginally born infants, with Staphylococcus species remaining significantly greater after 1 month [130]. This genus has been linked to obesity and increased energy intake, suggesting that infants born through C-section might be at risk of obesity [125,131,132]. Similarly, infants delivered via C-section showed lower α-diversity, which has been associated with a higher risk of developing obesity and type 2 diabetes [133,134]. After adjusting for potential confounders, infants born through C-section were found to be between 1.4 and 1.7 times more likely to develop childhood overweight or obesity [135,136].

## 7. Exposure of the Pregnant Woman to Toxic Substances in the Environment and the Risk of Obesity in Offspring

An endocrine-disrupting chemical (EDC) is a substance or a mixture of natural or man-made chemicals, exogenous to the organism, that possesses properties likely to interfere with the functions of the endocrine system, inducing an endocrine disruption with possible harmful effects on the health of an intact organism or its offspring. There is increasing evidence for the influence of environmental exposure to EDCs as a risk factor for obesity. In 2006, Blumberg and Grün [137] proposed the existence of “obesogens”, chemicals, including EDCs, as important but unsuspected players in the obesity pandemic. Numerous obesogens have been identified in recent years, and some of them could lead to transgenerational effects on obesity [138]. The “environmental obesogen hypothesis” underlies the notion that fetal exposure to certain EDCs makes affected individuals susceptible to the induction of fat mass and excess weight by modifying the epigenome of multipotent stromal stem cells, favoring them to the adipocyte lineage at the expense of bone [139,140]. However, human experimental studies investigating the associations of maternal toxicants with postnatal obesity, as compared to relatively large animal studies, remain limited and contradictory.

Phthalates and bisphenols are two classes of non-persistent EDCs that influence weight and adiposity and are ubiquitously found in personal care products, plastics, and other consumer products. It has been demonstrated that maternal weight gain during gestation can be affected by exposure to bisphenols and phthalates during the second trimester of pregnancy as a function of pre-pregnancy BMI. This suggests that women with higher pre-pregnancy BMI may be more susceptible to the obesogenic effects of phthalates [141]. Bisphenols and phthalates have also been shown to cross the placental barrier, resulting in direct fetal exposure. Although there are studies that have yielded conflicting results, others have demonstrated positive associations between maternal urinary total bisphenol and BPA (bisphenol a) concentrations in the second trimester of pregnancy and child weight between birth and 4 years of age and of average concentrations during pregnancy of various phthalate metabolites with child weight and BMI at 3 and 4 years of age [15]. An association has also been found between phthalate exposure in the first gestational trimester and a higher infant pericardial fat index at 10 years of age [142]. The relationship between prenatal exposure to three co-pollutants (phthalates, parabens, and phenols), measured by maternal prenatal urinary biomarkers, has also been analyzed for child adiposity at five years of age, showing consistent associations with both BMI z-score and overweight/obesity status [143].

Persistent organophosphate pesticides (OP) may also act as chemical obesogens and play an important role in the development of early-onset obesity. Results from prospective studies of maternal exposure to dichlorodiphenyldichloroethane (DDE) or dichlorodiphenyltrichloroethane (DDT) are conflicting in eliciting a positive association with obesity in offspring [144,145,146], but an overall positive association of combinations of persistent organic pollutants and maternal nutrients with risk of overweight/obesity has been demonstrated. Hexachlorobenzene and vitamin B12 are the biomarkers that contributed the most, supporting the hypothesis that nutritional status during pregnancy might modify the effect of environmental pollutants on child health, but further studies are needed to determine this [147].

## 8. Conclusions

In light of recent research findings, it is undeniable that a pregnant woman’s lifestyle impacts the metabolic health of her offspring. Although there is still a lack of sufficient scientific evidence, and some studies present conflicting results, certain indications suggest that aspects of a pregnant woman’s lifestyle—such as maintaining a healthy body weight and adhering to a balanced Mediterranean diet, which supports gut microbiota and reduces the risk of gestational diabetes, as well as avoiding exposure to obesogens — may significantly contribute to lowering the risk of obesity in the offspring.

Among the various maternal factors influencing fetal intrauterine programming, the maternal diet seems to be the primary focus of research. The Mediterranean dietary pattern, in contrast to a diet rich in pro-inflammatory, processed foods, is associated with a reduced risk of obesity in offspring, as it mitigates epigenetic mechanisms that promote excessive fat accumulation. The mother’s nutritional habits, body mass index (BMI), and hormonal changes during pregnancy, along with other factors such as the mode of delivery, may affect the composition of the placental gut microbiota, which colonizes the developing fetus. In cases of gut microbiota imbalance, known as dysbiosis, in the maternal body, inflammatory states and metabolic disturbances may arise, potentially programming an increased predisposition to obesity in children. This article also emphasizes that maternal exposure to obesogens and pregnancy complications, such as gestational diabetes, may further increase the risk of epigenetic modifications affecting fetal metabolism, which promotes the development of obesity.

Research into the relationship between obesity and epigenetics continues to provide significant insights, even though epigenetics is a relatively young field of science. One of the most well-understood epigenetic mechanisms, both at the whole-genome level and specific DNA loci, is DNA methylation. To date, research has identified the first epigenetic markers for obesity in the form of methylated DNA fragments detectable at birth. In the future, these markers may be used to predict obesity risk and plan strategies for its prevention and treatment. Recent studies suggest that prenatal exposure to environmental factors, including malnutrition, can lead to changes in DNA methylation that influence the manifestation of phenotypic traits in adulthood [148]. Adverse early-life conditions may have long-term health consequences, such as an increased risk of disease later in life. Furthermore, environmental exposures during critical developmental periods can alter the distribution of epigenetic markers, potentially promoting the development of obesity. Understanding these mechanisms will improve our ability to predict obesity risk in young people and develop effective prevention and treatment strategies. Additionally, some epigenetic markers can be influenced by both prenatal factors and lifestyle changes in adulthood, offering opportunities for postnatal interventions to mitigate adverse changes in the epigenomic profile [148].

With the increasing availability and decreasing cost of advanced sequencing technologies offering higher coverage, we anticipate new discoveries in epigenetics. These advancements may enhance our understanding of the complex etiology of obesity and other diseases, enabling the development of more effective early prevention and treatment strategies. Recent findings underscore the importance of epigenetic programming in obesity and its comorbidities, which could open new therapeutic pathways. However, much of the human epigenome remains unexplored. Epigenetic research faces greater challenges than genetic studies because the epigenome is specific to particular cells and tissues and changes over time. To effectively treat and prevent childhood obesity, we must fully understand the genetic, epigenetic, and metabolic mechanisms that contribute to it. Further high-quality research is needed to understand better the metabolic and epigenetic relationships in the context of maternal factors that predispose offspring to obesity. The findings from future studies may be crucial in designing strategies for the prevention of childhood obesity. Additionally, it is important to note that the studies discussed in this article address only some of the lifestyle factors of pregnant women that may affect the metabolic health of the offspring. Some research suggests that physical activity, sleep, and other lifestyle aspects also play a crucial role in the metabolic and epigenetic programming of the child’s health, offering protection against obesity [10,149,150,151,152]. Moreover, numerous studies indicate that the preconception period, including a woman’s preparation for pregnancy, as well as the lifestyle and diet of the father, significantly influence the child’s predisposition to excessive fat accumulation [153,154,155].

## Figures and Tables

**Table 1:** Characteristics of the main types of epigenetic modifications [42].

Process	Mechanism	Effect
DNA methylation	Addition of a methyl group to CpG sites	Depending on the localization of CpGs: silencing of gene expression or activation of gene transcription.
Post-translational modifications of histones	Chemical modification of histone proteins (e.g., methylation, acetylation, phosphorylation)	Alteration of chromatin structure and regulation of the binding of chromatin-associated proteins. Depending on the type of modification: activation or repression of transcription.
Non-coding RNAs	Regulation of gene expression through short ncRNAs (miRNA, piRNA, and siRNA) and long ncRNAs (eRNA, lincRNA, and lncRNA)	Involvement in epigenetic regulation at transcriptional, translational, and post-translational levels, including, e.g., chromatin remodeling and RNA interference.

**Table 2:** Main characteristics of the Western and Mediterranean diets [87].

Western Diet	Mediterranean Diet
Pre-packaged food	High intake of fruits and vegetables
Red and processed meat	Whole grains
Refined grains	Legumes
High-sugar drinks	Olive oil
Foods with added sugar (pastries, candy, sweets, etc.)	Nuts
High intake of trans/saturated fats (meat, butter, fried foods)	Moderate intake of animal protein

## Data Availability

Not applicable.
